# Supplementary material for: VAMP7-dependent late endosomal secretion of ER and mitochondrial proteins impacts the tumor microenvironment and macrophage engagement
Source: Nat Commun. 2026 Feb 21;17:3012. doi: 10.1038/s41467-026-69900-4 (PMC13035943; doi:10.1038/s41467-026-69900-4)
Supplement: Supplementary file 1 — Supplementary Information File [file 41467_2026_69900_MOESM1_ESM.pdf]

**Supplementary Information for VAMP7-dependent late endosomal secretion of ER and mitochondrial proteins impacts the tumor microenvironment and macrophage engagement**

**Supplementary Figures 1-7**

**Supplementary Figure 1.** VAMP7KO impairs the secretion of tubular ER and mitochondrial proteins.

**Supplementary Figure 2.** Degradative autophagy in NRK cells.

**Supplementary Figure 3.** RNA-seq analysis of VAMP7KO and ATG5KO NRK datasets.

**Supplementary Figure 4.** VAMP7KO and ATG5KO NRK have an increased mitochondrial mass but decreased mitochondrial efficiency.

**Supplementary Figure 5.** VAMP7KO and ATG5KO NRK have an alteration in the mitochondrial structure.

**Supplementary Figure 6.** The interaction of CD63 with RTN3 and of VAMP7 with its partner SNAREs.

**Supplementary Figure 7.** VAMP7 in primary brain tumors.

**Supplementary Methods**

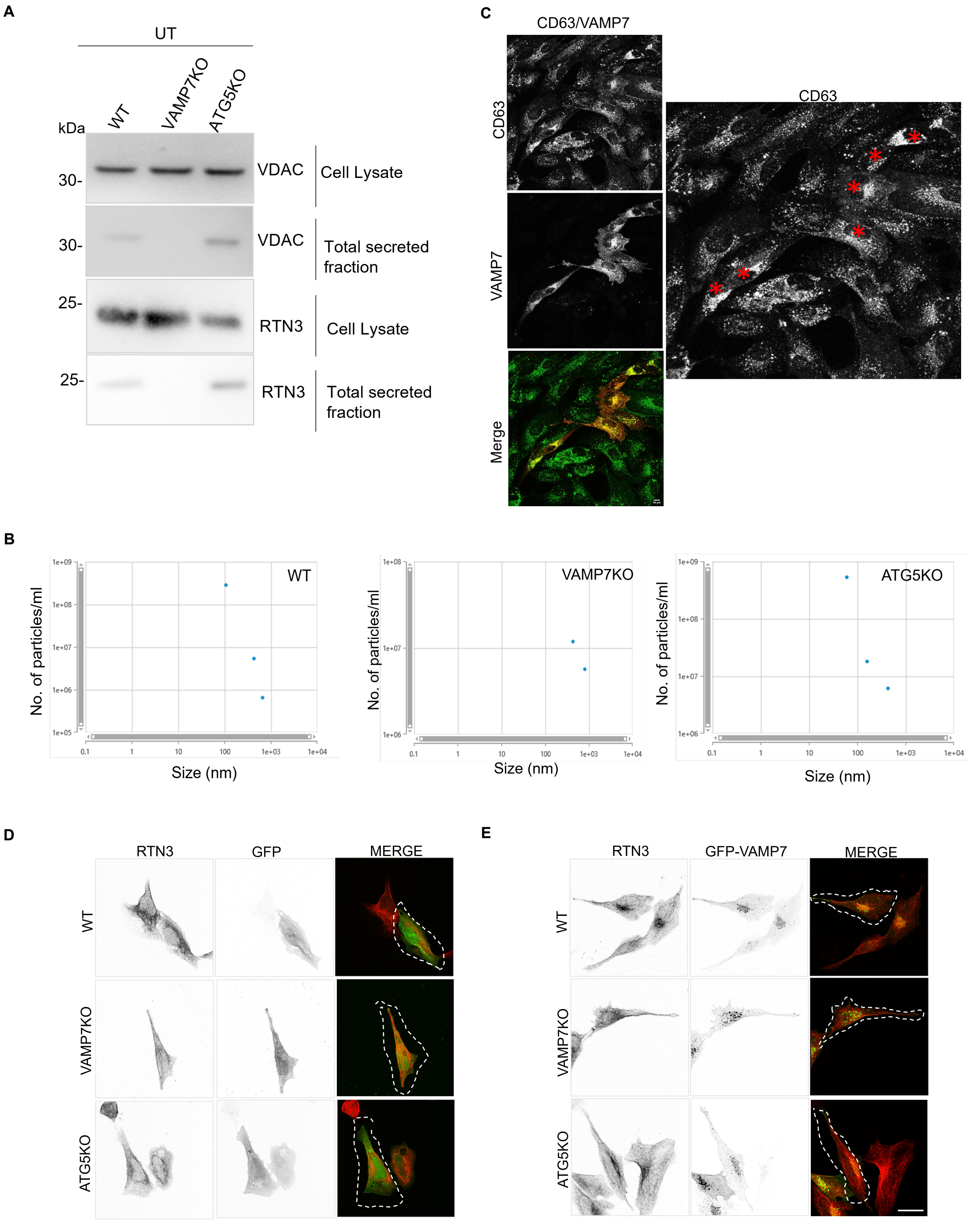

A
UT
kDa
WT
VAMP7KO
ATG5KO
30-
VDAC
Cell Lysate
30-
VDAC
Total secreted fraction
25-
RTN3
Cell Lysate
25-
RTN3
Total secreted fraction
C
CD63/VAMP7
CD63
VAMP7
Merge
CD63
B
No. of particles/ml
Size (nm)
WT
VAMP7KO
ATG5KO
D
RTN3
GFP
MERGE
WT
VAMP7KO
ATG5KO
E
RTN3
GFP-VAMP7
MERGE
WT
VAMP7KO
ATG5KO

**Supplementary Figure 1: VAMP7KO impairs the secretion of tubular ER and mitochondrial proteins. (A)** WT, VAMP7KO, and ATG5KO NRK cells were serum starved overnight. The next day, the secreted media was processed, and the total secreted protein was precipitated using acetone. The total secreted fraction and cell lysates were collected and analyzed for secreted RTN3 and VDAC. The experiment was repeated thrice independently with similar results. **(B)** WT, VAMP7KO and ATG5KO NRK cells were serum starved overnight. The next day, the secreted media was processed using a commercial kit, and exosomes were isolated. The properties and numbers of the secreted extracellular vesicles from WT, VAMP7KO, and ATG5KO NRK cells were analyzed using DLS Zetasizer. Note the virtual disappearance of small EVs ~100nm size in VAMP7KO secretome. The experiment was repeated thrice independently with similar results. **(C)** WT and VAMP7KO cells were either left untransfected or were transfected with pcDNA-VAMP7 and immunostained for VAMP7 and CD63. The red stars mark the cells in which VAMPKO has been rescued. Note the perinuclear accumulation of CD63 in these cells. **(D,E)** WT, VAMP7KO and ATG5KO NRK cells were either left untransfected or were transfected with GFP (control) or GFP-VAMP7 and immunostained with RTN3.The experiment was repeated thrice independently with similar results.

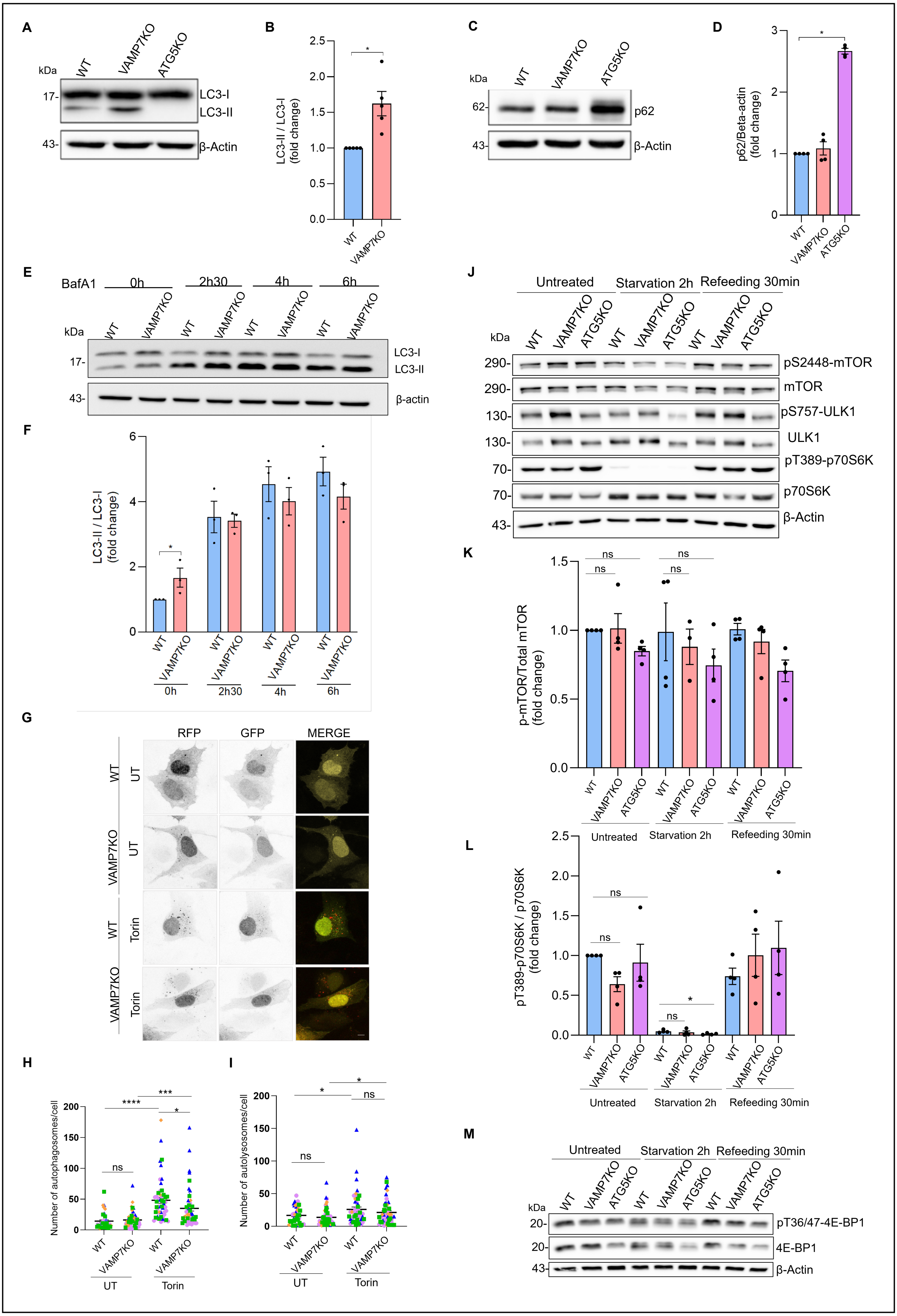

A
kDa
WT
VAMP7KO
ATG5KO
17-
LC3-I
LC3-II
43-
β-Actin
B
LC3-II / LC3-I (fold change)
WT
VAMP7KO
C
62-
p62
D
p62/Beta-actin (fold change)
E
BafA1
0h
2h30
4h
6h
LC3-I
LC3-II
β-actin
F
LC3-II / LC3-I (fold change)
G
RFP
GFP
MERGE
UT
Torin
H
Number of autophagosomes/cell
UT
Torin
I
Number of autolysosomes/cell
J
Untreated
Starvation 2h
Refeeding 30min
290-
pS2448-mTOR
mTOR
130-
pS757-ULK1
ULK1
70-
pT389-p70S6K
p70S6K
K
p-mTOR/Total mTOR (fold change)
L
pT389-p70S6K / p70S6K (fold change)
M
20-
pT36/47-4E-BP1
4E-BP1
ns

**Supplementary Figure 2: Degradative autophagy in NRK cells. (A-D)** WT, VAMP7KO and ATG5KO NRK cell lysates were blotted for LC3 and p62 expression. The fold change ratio of LC3-II/LC3-I and p62/beta-Actin was quantified and plotted (Mean with SEM). n=4 independent experiments; either One sample t-test (for LC3II/LC3-I) or Kruskal-Wallis test (for p62) were used to determine statistical significance, * p-val < 0.05. **(E, F)** BafA1 treated WT, VAMP7KO and ATG5KO NRK cell lysates were blotted for LC3 expression. The fold change ratio of LC3-II/LC3-I was quantified and plotted (Mean with SEM). n=3 independent experiments; One sample t-test (LC3II/LC3-I) was used to determine statistical significance, * p-val < 0.05. **(G-I)** WT, VAMP7KO and ATG5KO NRK cells were transfected with RFP-GFP-LC3. 36 hours after transfection the cells were either left untreated or treated with Torin1 (500nM) for 2hours, fixed, processed and imaged by confocal microscopy, Scale bar = 25 µm. The number of autophagosomes and autolysosomes was counted using the 'cell counter' plugin of FIJI/ImageJ and plotted as Mean with SEM. n=3 independent experiments; Welch's t-test (two-tailed) was used to determine the statistical significance,* p-val < 0.05, ns: non-significant. **(J-M)** WT, VAMP7KO and ATG5KO NRK cell lysates were either left untreated, starved for 2 hours in EBSS, or starved for 2 hours and refed for 30 minutes. The lysates were blotted for phosphorylated and total mTOR and its downstream effectors phosphorylated p70S6 kinase and 4EBP1. The fold change ratio was quantified and plotted (Mean with SEM). n=4 independent experiments; Kruskal-Wallis test was used to determine the statistical significance, **** p-val < 0.0001, *** p-val < 0.001, * p-val < 0.05, ns: non-significant.

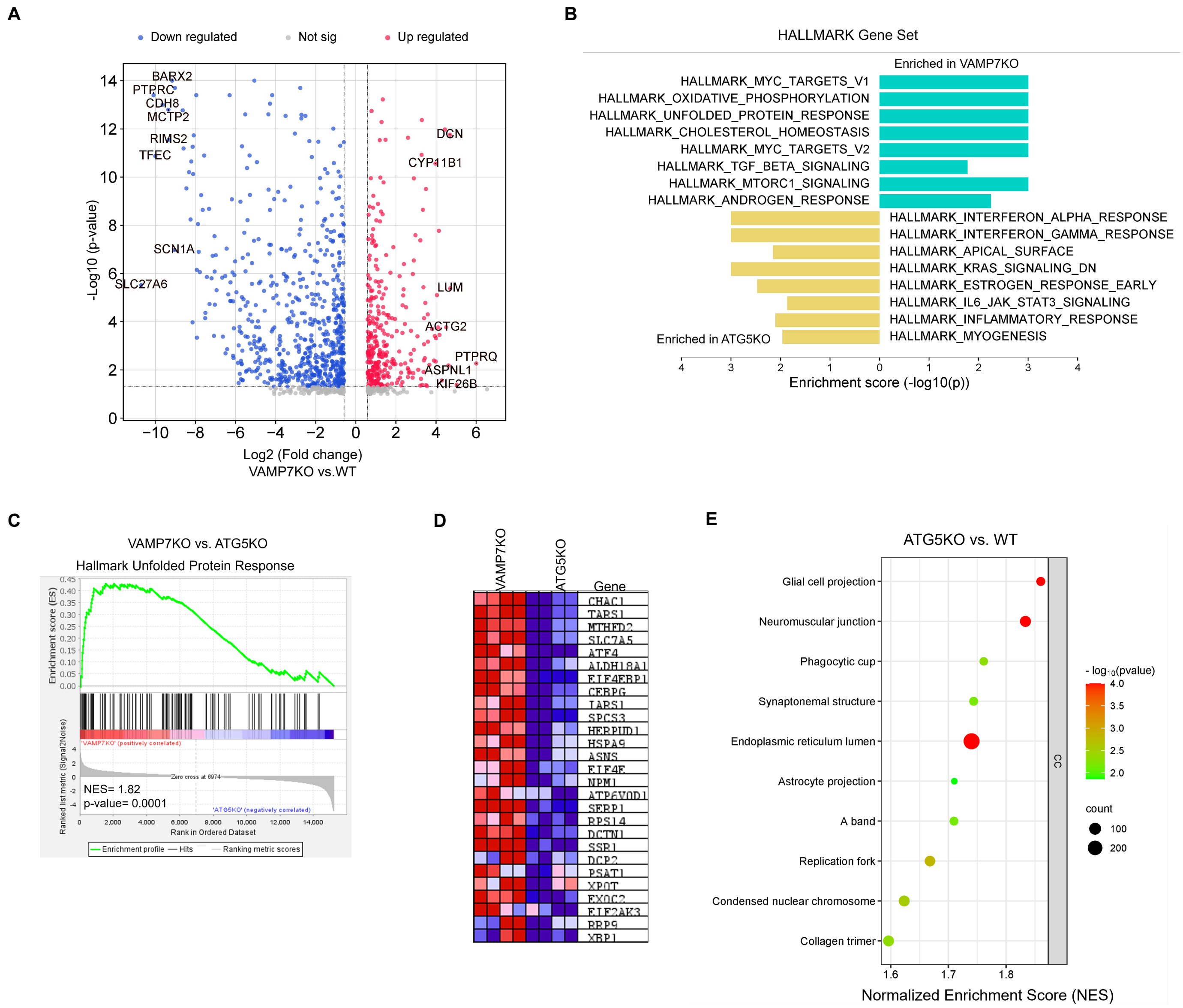

A
Down regulated
Not sig
Up regulated
BARX2
PTPRC
CDH8
MCTP2
RIMS2
TFEC
SCN1A
SLC27A6
DCN
CYP11B1
LUM
ACTG2
PTPRQ
ASPNL1
KIF26B
-Log10 (p-value)
Log2 (Fold change)
VAMP7KO vs.WT
B
HALLMARK Gene Set
Enriched in VAMP7KO
HALLMARK_MYC_TARGETS_V1
HALLMARK_OXIDATIVE_PHOSPHORYLATION
HALLMARK_UNFOLDED_PROTEIN_RESPONSE
HALLMARK_CHOLESTEROL_HOMEOSTASIS
HALLMARK_MYC_TARGETS_V2
HALLMARK_TGF_BETA_SIGNALING
HALLMARK_MTORC1_SIGNALING
HALLMARK_ANDROGEN_RESPONSE
HALLMARK_INTERFERON_ALPHA_RESPONSE
HALLMARK_INTERFERON_GAMMA_RESPONSE
HALLMARK_APICAL_SURFACE
HALLMARK_KRAS_SIGNALING_DN
HALLMARK_ESTROGEN_RESPONSE_EARLY
HALLMARK_IL6_JAK_STAT3_SIGNALING
HALLMARK_INFLAMMATORY_RESPONSE
HALLMARK_MYOGENESIS
Enriched in ATG5KO
Enrichment score (-log10(p))
C
VAMP7KO vs. ATG5KO
Hallmark Unfolded Protein Response
Enrichment score (ES)
Ranked list metric (Signal2Noise)
'VAMP7KO' (positively correlated)
Zero cross at 6974
'ATG5KO' (negatively correlated)
NES= 1.82
p-value= 0.0001
Rank in Ordered Dataset
Enrichment profile
Hits
Ranking metric scores
D
VAMP7KO
ATG5KO
Gene
CHAC1
TARS1
MTHFD2
SLC7A5
ATF4
ALDH18A1
EIF4EBP1
CEBPG
IARS1
SPCS3
HERPUD1
HSPA9
ASNS
EIF4E
NPM1
ATP6V0D1
SEPP1
RPS14
DCTN1
SSR1
DCP2
PSAT1
XPOT
EXOC2
EIF2AK3
PRP9
XBP1
E
ATG5KO vs. WT
Glial cell projection
Neuromuscular junction
Phagocytic cup
Synaptonemal structure
Endoplasmic reticulum lumen
Astrocyte projection
A band
Replication fork
Condensed nuclear chromosome
Collagen trimer
CC
- log10(pvalue)
count
Normalized Enrichment Score (NES)

**Supplementary Figure 3**: **RNA-seq analysis of VAMP7KO and ATG5KO NRK datasets.** **(A)** Volcano plot of transcriptomic analyses showing differentially expressed genes between VAMP7KO and WT NRK cells. n=4 independent experiments. The entire list of diiferently expressed genes in VAMP7KO and ATG5KO NRK are provided as supplementary files. **(B)** Gene set enrichment analysis results for hallmark gene sets comparing VAMP7KO and ATG5KO datasets. Whole genes ordered by the z-scores from the meta-analysis were used as ranked genes for analysis. The top positively enriched biological pathways from Gene Set Enrichment Analysis (GSEA) hallmark gene sets for VAMP7KO dataset are depicted in light blue. In contrast, the hallmark gene sets enriched in the ATG5KO dataset are displayed in dark yellow. Note the enrichment of the expression of genes related to UPR. **(C)** GSEA plot for the Hallmark Unfolded Protein Response geneset shows a significant enrichment in the VAMP7KO dataset, Normalized enrichment score (NES)=1.82. Positive NES values indicate enrichment among upregulated genes and negative values indicate enrichment among downregulated genes. **(D)** Heatmap showing the respective transcripts levels (fold change) associated to unfolded protein response in VAMP7KO versus ATG5KO NRK cells obtained from GSEA analysis. n=4 independent experiments, red indicates positive enrichment and blue indicates negative enrichment. **(E)** GSEA performed for Gene Ontology (GO) pathways relative to Cellular Components (CC) shows a significant enrichment of ER-lumen proteins in the ATG5KO dataset. p-values were determined according to analysis in the GSEA database for annotation, visualization, and enrichment analysis (Fisher exact P-value). X-axis shows NES values from GSEA, indicating the strength of pathway enrichment in ATG5KO relative to WT, Y-axis Gene represents Ontology cellular component (GO-CC) terms.

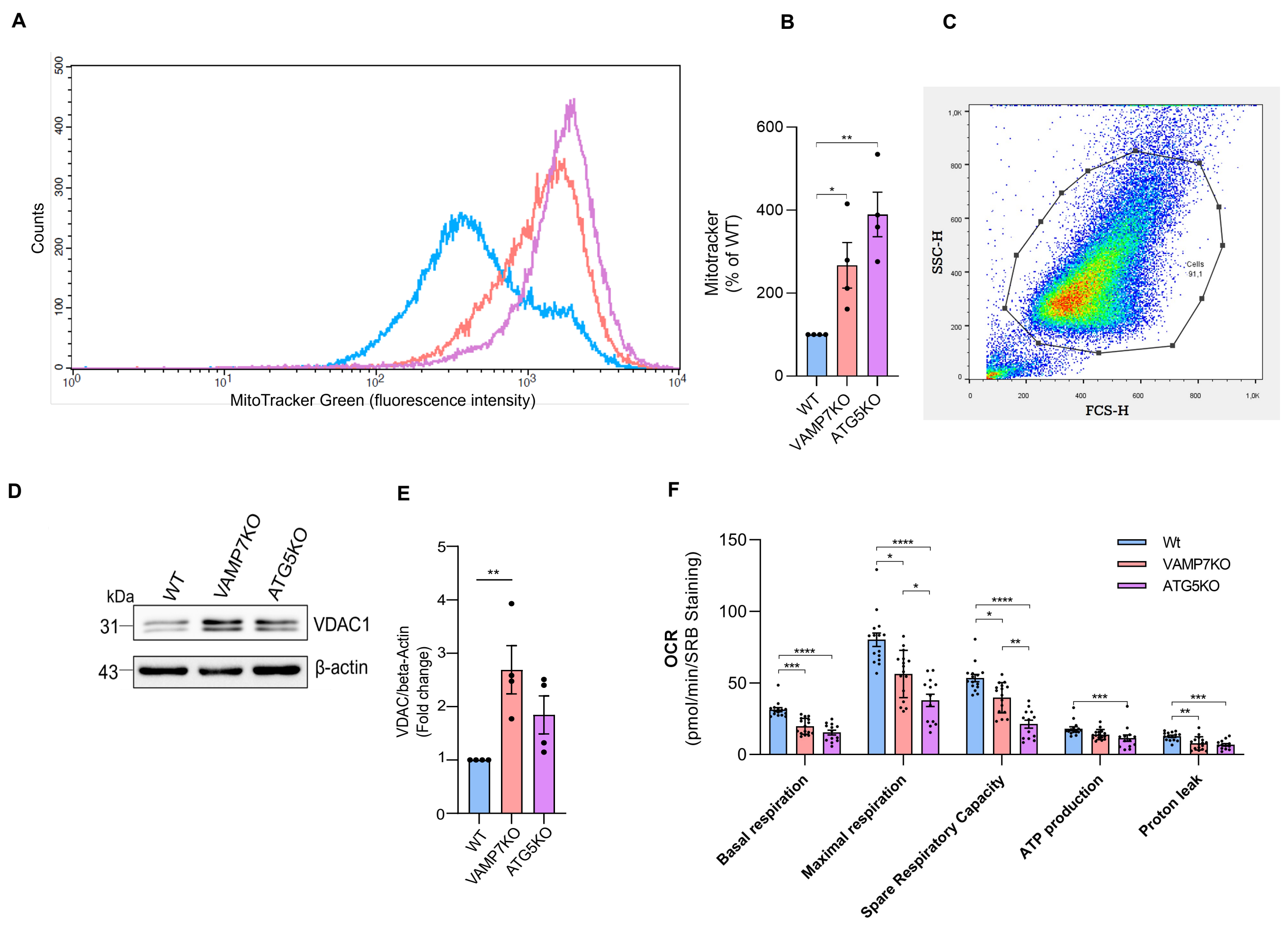
A
Counts
MitoTracker Green (fluorescence intensity)
B
Mitotracker (% of WT)
WT
VAMP7KO
ATG5KO
C
SSC-H
FCS-H
Cells 91,1
D
kDa
WT
VAMP7KO
ATG5KO
31
VDAC1
43
β-actin
E
VDAC/beta-Actin (Fold change)
F
OCR (pmol/min/SRB Staining)
Wt
VAMP7KO
ATG5KO
Basal respiration
Maximal respiration
Spare Respiratory Capacity
ATP production
Proton leak

**Supplementary Figure 4: VAMP7KO and ATG5KO NRK have an increased mitochondrial mass but decreased mitochondrial efficiency. (A, B)** WT, VAMP7KO, and ATG5KO NRK cells were stained with Mitotracker Green and fluorescence was quantified by flow cytometry. Increased fluorescence is associated with increased mitochondrial mass. On the left, an example of a typical histogram for MitoTracker-dependent fluorescence for the three cell lines, while on the right the graph shows the quantification of mean fluorescence intensity for WT and KO cells (Mean with SEM). n=3 independent experiments; Kruskal-Wallis test was used to determine the statistical significance WT and KO samples, * p-val < 0.05 and ** p-val < 0.01. **(C)** FCS-H vs SSC-H plots were used to define the gating, thereby excluding debris and very big cells. **(D, E)** WT, VAMP7KO and ATG5KO NRK cell lysates were blotted for VDAC expression. The fold change ratio of VDAC/beta-Actin was quantified and plotted (Mean with SEM). n=3 independent experiments; Kruskal-Wallis test was used to determine the statistical significance between WT and KO samples. ** p-val < 0.01. (**F**) Oxygen consumption rate (OCR) is significantly lower in VAMP7KO and ATG5KO cells than in WT cells, indicating respiratory chain inefficiency. n=3 independent experiments; Kruskal-Wallis test was used to determine statistical significance, * p-val < 0.05, ** p-val < 0.01, *** p-val < 0.001, **** p-val < 0.0001.

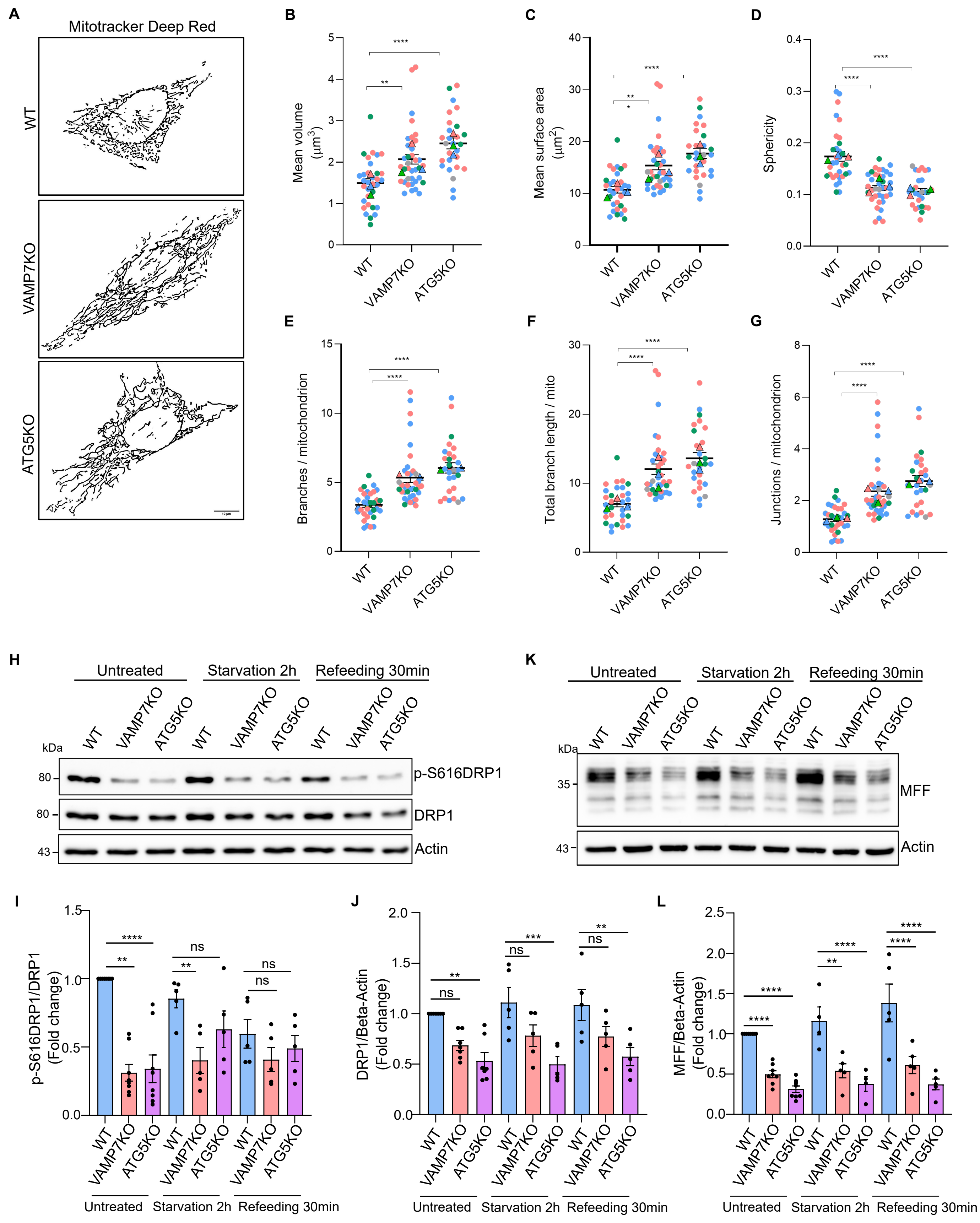
A
Mitotracker Deep Red
WT
VAMP7KO
ATG5KO
B
Mean volume (μm³)
C
Mean surface area (μm²)
D
Sphericity
E
Branches / mitochondrion
F
Total branch length / mito
G
Junctions / mitochondrion
H
Untreated
Starvation 2h
Refeeding 30min
kDa
p-S616DRP1
DRP1
Actin
K
MFF
I
p-S616DRP1/DRP1 (Fold change)
J
DRP1/Beta-Actin (Fold change)
L
MFF/Beta-Actin (Fold change)
ns

**Supplementary Figure 5: VAMP7KO and ATG5KO NRK have an alteration in the mitochondrial structure. (A)** WT, VAMP7KO and ATG5KO NRK cells were stained with Mitotracker Deep Red and subsequently imaged by confocal microscopy for 3D mitochondrial network analyses. Both morphological parameters **(B-D)** and connectivity parameters **(E-G)** show that VAMP7KO and ATG5KO NRK cells exhibit larger, more branched mitochondria than WT cells, supporting a more pro-fusion mitochondrial environment. Data is represented as Mean with SEM from n=3 independent experiments; Kruskal-Wallis followed by post hoc Dunn's test was used to determine statistical significance, ** p-val < 0.01, *** p-val < 0.001 and **** p-val < 0.0001. Scale bar = 10 μm. **(H-J)** WT, VAMP7KO and ATG5KO NRK cell lysates were either left untreated, starved for 2 hours in EBSS, or starved for 2 hours and refed for 30 minutes. The lysates were blotted for pS616-DRP1 and DRP1. The fold change ratios of pS616-DRP1/DRP1 and DRP1/β-Actin were quantified and plotted (Mean with SEM), showing a decrease in DRP1 total protein levels and an even stronger decrease in the pro-fission phosphorylation at S616 in VAMP7KO and ATG5KO. n=3 independent experiments; One-way ANOVA with Bonferroni's post hoc test was used to determine the statistical significance, ** p-val < 0.01, *** p-val < 0.001, **** p-val < 0.0001, ns: non-significant. **(K, L)** WT, VAMP7KO and ATG5KO NRK cell lysates were blotted for MFF protein levels. The fold change ratio of MFF/β-Actin was quantified and plotted (Mean with SEM), showing a strong decrease in MFF levels, the main mitochondrial fission initiator, in both KO cell lines. n=3 independent experiments; One-way ANOVA with Bonferroni's post hoc test was used to determine the statistical significance, ** p-val < 0.01, **** p-val < 0.0001.

A

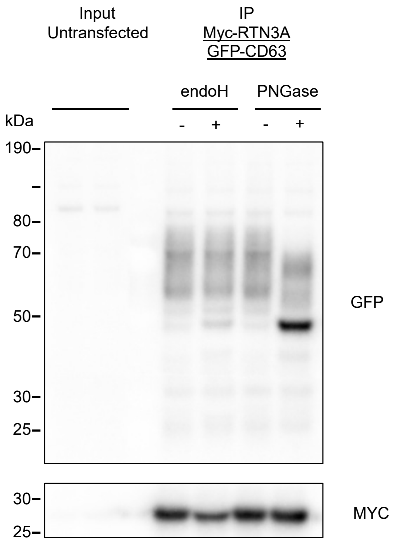


B

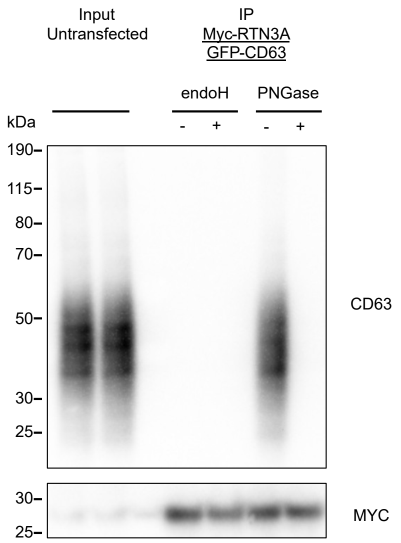


C

| Function | Protein | Bait |
|---|---|---|
| Membrane fusion | Syntaxin7 | VAMP7 |
| Membrane fusion | Syntaxin8 | VAMP7 |
| Membrane fusion | SNAP23 | VAMP7 |
| Membrane fusion | SNAP29 | VAMP7 |
| Membrane fusion | Vti1B | VAMP7 |
| Membrane fusion | SNAP47 | VAMP7 |
| Membrane fusion | Syntaxin16 | VAMP7 |
| Membrane fusion | Syntaxin12 | VAMP7 |
| Membrane fusion | Vti1A | VAMP7 |
| Membrane fusion | Syntaxin6 | VAMP7 |
| Membrane fusion | Syntaxin5 | VAMP7 |
| Membrane fusion | Syntaxin17 | VAMP7 |
| Membrane fusion | Syntaxin4 | VAMP7 |
| Late endosome trafficking/ extracellular vesicle generation | CD63 | VAMP7 |
| Vesicle trafficking | Rab21 | VAMP7 |
| Membrane fusion | Syntaxin18 | VAMP7 |

D

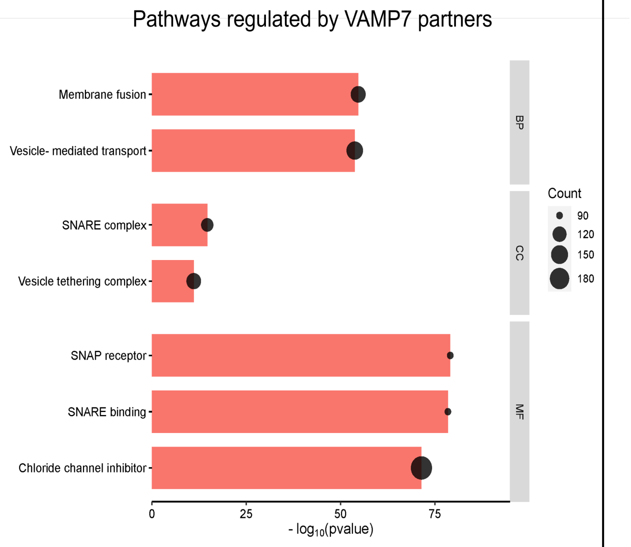


E

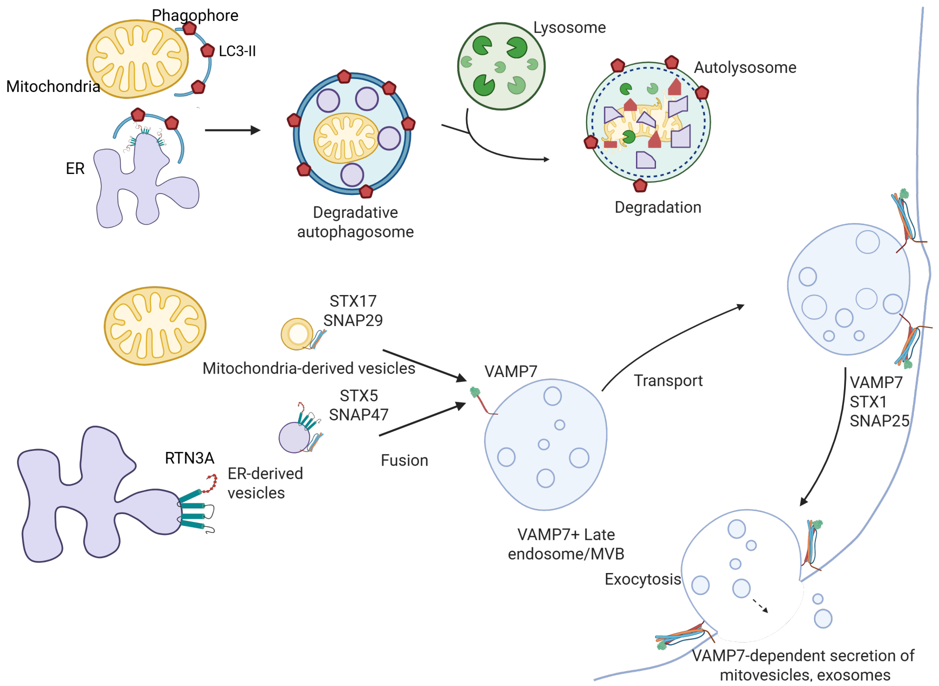

**Supplementary Figure 6: The interaction of CD63 with RTN3 and of VAMP7 with its partner SNAREs. (A, B)** WT NRK were either left untransfected or transfected with a combination of Myc-RTN3A and GFP-CD63. Cell lysates were either left untreated or treated with Endo H or PNGase F (Rapid PNGase F) then blotted for GFP or CD63. Following the manufacturer's protocol for Endo H treatment, a pre-denaturating step with boiling at 100°C was realized even in control condition (-Endo H). GFP signal was not impacted by this step whereas CD63 signal completely disappeared because AD1 anti-rat CD63 antibody only recognizes the native conformation of CD63. Myc signal is not affected by the pre-denaturing step. The experiment was repeated thrice independently with similar results. **(C)** List of SNAREs and membrane trafficking proteins significantly enriched in four independent replicates of WT NRK cells transfected with GFP-VAMP7 vs. the background (empty GFP); n=4 independent experiments **(D)** Gene Ontology analysis shows that most enriched proteins in the proteome have a SNARE motif and are involved in vesicle mediated transport. **(E)** Graphical abstract illustrating our proposed model. Autophagy (top) and late endosomal secretion (bottom) act in parallel to handle cargo derived from mitochondria and the ER. VAMP7 drives the fusion of mitochondria- and ER-originating vesicles with late endosomes, which then merge with the plasma membrane to release their content via extracellular vesicles. Created in BioRender. Galli, T. (2026) https://BioRender.com/o21m075.

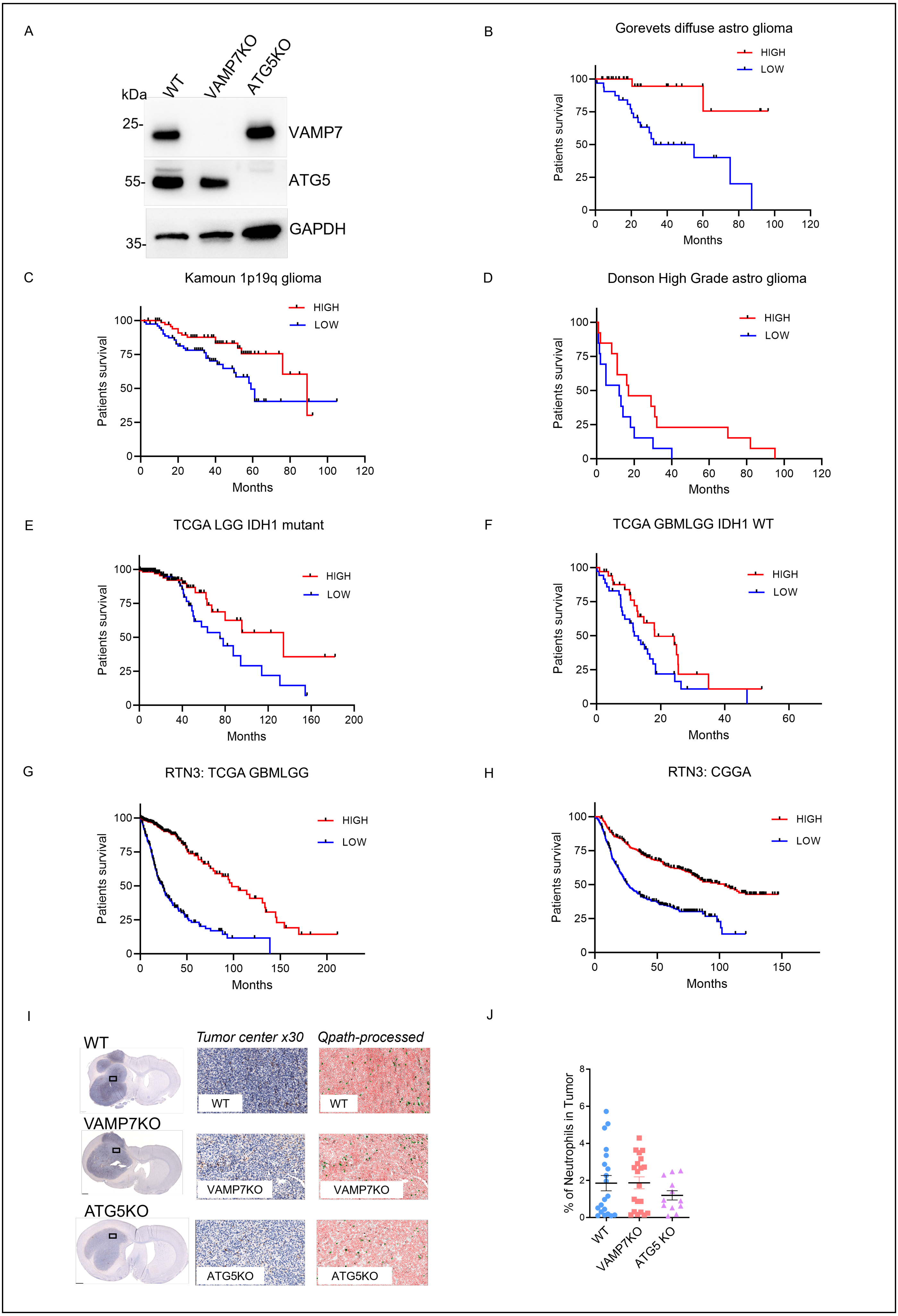
A
kDa
WT
VAMP7KO
ATG5KO
25-
55-
35-
VAMP7
ATG5
GAPDH
B
Gorevets diffuse astro glioma
HIGH
LOW
Patients survival
Months
C
Kamoun 1p19q glioma
D
Donson High Grade astro glioma
E
TCGA LGG IDH1 mutant
F
TCGA GBMLGG IDH1 WT
G
RTN3: TCGA GBMLGG
H
RTN3: CGGA
I
WT
VAMP7KO
ATG5KO
Tumor center x30
Qpath-processed
J
% of Neutrophils in Tumor
WT
VAMP7KO
ATG5 KO

**Supplementary Figure 7: VAMP7 in primary brain tumors. (A)** WT, VAMP7KO and ATG5KO RG2 cell lysates were blotted for VAMP7, ATG5 and GAPDH to confirm the KOs of VAMP7 and ATG5 in these cells. **(B-F)** Kaplan-Meier survival curve of patients bearing VAMP7 *low* (blue) or *high* (red) expression in different cohorts of primary brain tumors with specific characteristics. Log-rank p-val = 0.0007 **(B)**; 0.05 **(C)**; 0.06 **(D)**; 0.05 **(E)**; 0.05 **(F)**. (**G-H**) Kaplan-Meier survival curves for patients with RTN3 low (blue) or high (red) expression across different cohorts of primary brain tumors with specific characteristics. Log-rank p-val = 0 for both TCGA-GBMLGG and CCGA. **(I)** Tumor sections were double-stained with HES and anti-MPO antibodies (neutrophils) and revealed with secondary antibodies coupled to HRP and DAB. Magnification of the tumor center and Qpath analyses are shown for tumors derived from WT, VAMP7KO or ATG5KO cells**. (J)** Quantitation of neutrophil staining in the tumor center. n=3 independent experiments; Welch's t-test (two-tailed) was used to determine the statistical significance, ns=non-significant.

**Supplementary Methods**

*Reverse transcription on RG2 cells*

All heating steps were done in PCR machine in "Incubate mode". A control (-) was performed: same steps but without addition of the enzyme.

Mixtures were prepared with 250 ng of "Random primer" from Sigma-Aldrich (58875), 500 ng of mRNA preparation, 5 mM dNTP Mix and qsp distilled water. Mixtures were heated to 65°C for 5 min then quick chilled on ice. Contents were collected after brief centrifugation and 5X First-Strand Buffer and 0.1M DTT were added. Contents were gently mixed and incubated at 25°C for 2 min. 1 µl (200 units) of SuperScript II Reverse Transcriptase was added and mixtures were incubated at 25°C for 10 min. Then, new incubation was realized at 42°C for 50 min. The reaction was inactivated by heating at 70°C for 15 min.

The following primers were used:

| | IDH-1 rat genomic/RT for amplification and sequencing | |
|---|---|---|
| Sebn195 | CTATGACTTAGGCATAGAG | Fwd |
| Sebn196 | TTGGTCCCCATAGG | Rev |

| | IDH-2 rat genomic/RT for amplification and sequencing | |
|---|---|---|
| Sebn197 | AGTTCAAGCTGAAGAAAATG | Fwd |
| Sebn198 | CTGGTCGCCATGGG | Rev |

RT PCR for IDH1/2 was performed following this program:

| Step | Temperature | Time | Number of cycles |
|---|---|---|---|
| Initial denaturation | 98°C | 5 min | 1 cycle |
| Denaturation | 98°C | 10 sec | 35 cycles |
| Hybridation | 56°C/59°C | 30 sec | |
| Elongation | 72°C | 15 sec/30 sec | |
| Final elongation | 72°C | 5 min | 1 cycle |
| Storage | 15°C | 10 min | 1 cycle |
