# Supplementary material for: VAMP7-dependent late endosomal secretion of ER and mitochondrial proteins impacts the tumor microenvironment and macrophage engagement
Source: Nat Commun. 2026 Feb 21;17:3012. doi: 10.1038/s41467-026-69900-4 (PMC13035943; doi:10.1038/s41467-026-69900-4)
Supplement: Supplementary file 2 — Description of Additional Supplementary File [file 41467_2026_69900_MOESM2_ESM.pdf]

Description of Additional Supplementary Files

Supplementary Data 1: A list of Differentially expressed genes in the VAMP7KO versus WT datasets.

Supplementary Data 2: A list of Differentially expressed genes in the ATG5KO versus WT datasets.

Supplementary Data 3: List of VAMP7 interacting partners identified by Co-IP proteomics

Supplementary Data 4: mRNA sequencing of IDH1 and IDH2 in RG2 cells demonstrating the expression of the WT forms
