# Supplementary material for: VAMP7-dependent late endosomal secretion of ER and mitochondrial proteins impacts the tumor microenvironment and macrophage engagement
Source: Nat Commun. 2026 Feb 21;17:3012. doi: 10.1038/s41467-026-69900-4 (PMC13035943; doi:10.1038/s41467-026-69900-4)
Supplement: Supplementary file 7 — Reporting summary [file 41467_2026_69900_MOESM7_ESM.pdf]

nature portfolio

Corresponding author(s): Thierry Galli

Last updated by author(s): Jan 27, 2026

# Reporting Summary

Nature Portfolio wishes to improve the reproducibility of the work that we publish. This form provides structure for consistency and transparency in reporting. For further information on Nature Portfolio policies, see our Editorial Policies and the Editorial Policy Checklist.

## Statistics

For all statistical analyses, confirm that the following items are present in the figure legend, table legend, main text, or Methods section.

| n/a | Confirmed | |
|---|---|---|
| ☐ | ☒ | The exact sample size (*n*) for each experimental group/condition, given as a discrete number and unit of measurement |
| ☐ | ☒ | A statement on whether measurements were taken from distinct samples or whether the same sample was measured repeatedly |
| ☐ | ☒ | The statistical test(s) used AND whether they are one- or two-sided<br>*Only common tests should be described solely by name; describe more complex techniques in the Methods section.* |
| ☒ | ☐ | A description of all covariates tested |
| ☐ | ☒ | A description of any assumptions or corrections, such as tests of normality and adjustment for multiple comparisons |
| ☐ | ☒ | A full description of the statistical parameters including central tendency (e.g. means) or other basic estimates (e.g. regression coefficient) AND variation (e.g. standard deviation) or associated estimates of uncertainty (e.g. confidence intervals) |
| ☐ | ☒ | For null hypothesis testing, the test statistic (e.g. *F*, *t*, *r*) with confidence intervals, effect sizes, degrees of freedom and *P* value noted<br>*Give P values as exact values whenever suitable.* |
| ☒ | ☐ | For Bayesian analysis, information on the choice of priors and Markov chain Monte Carlo settings |
| ☒ | ☐ | For hierarchical and complex designs, identification of the appropriate level for tests and full reporting of outcomes |
| ☐ | ☒ | Estimates of effect sizes (e.g. Cohen's *d*, Pearson's *r*), indicating how they were calculated |

*Our web collection on statistics for biologists contains articles on many of the points above.*

## Software and code

Policy information about availability of computer code

| | |
|---|---|
| Data collection | Zeiss Confocal Microscope LSM 880 and LSM 980 with Airyscan 2, Zeiss LSM 710 Confocal Laser Scanning microscope, Leica SP5 Confocal, Leica SP8 STED 3DX, BD FACS Calibur TM (BD Biosciences), Seahorse XF96 Extracellular Flux Analyzer (Agilent), Seahorse XF Analyzer (XFp, Proteigene/ Agilent), Discovery XT machine (Roche), Nanozoomer 2.0 RS Hamamatsu, DLS Zetasizer (Malvern Instruments), Chemidoc Imager TM (Biorad), HPLC (Bruker Daltonics, Germany), Transmission Electron Microscope (Tecnai Spirit G2; ThermoFischer, Eindhoven), Illumina NextSeq 2000 sequencer. |
| Data analysis | RTA version 2.7.7 and BCL Convert version 3.8.4 (Illumina), cutadapt version 4.2, DESeq2 1.34.0, iDEP, SRplot, Graphpad Prism v10.6.1, Gene Set Enrichment Analysis (GSEA), Qpath software, Fiji/ImageJ (NIH), Python Integrated Development Environment (IDE)-Spyder software, FlowJO, Perseus software (version 1.6.15), Icy software, Analyze-measure, Mitochondria Analyzer, Cell counter, Jacop and Sphere plugins in Fiji/ImageJ. The script for the Sphere plugin has been deposited in Zenodo under the following DOI https://doi.org/10.5281/zenodo.17473585. |

For manuscripts utilizing custom algorithms or software that are central to the research but not yet described in published literature, software must be made available to editors and reviewers. We strongly encourage code deposition in a community repository (e.g. GitHub). See the Nature Portfolio guidelines for submitting code & software for further information.

## Data

Policy information about availability of data

All manuscripts must include a data availability statement. This statement should provide the following information, where applicable:

- Accession codes, unique identifiers, or web links for publicly available datasets
- A description of any restrictions on data availability
- For clinical datasets or third party data, please ensure that the statement adheres to our policy

All data associated with this study can be found in the paper, the Supplementary materials, and the Source data file. Research materials are available upon request. The mass spectrometry proteomics data have been deposited to the ProteomeXchange Consortium via the PRIDE partner repository with the dataset identifier PXD057152. The RNA sequencing data have been deposited to the GEO repository with the dataset identifier GSE280209. The script for the Sphere plugin has been deposited in Zenodo under the following DOI: https://doi.org/10.5281/zenodo.17473585. Source data are provided with this paper.

## Research involving human participants, their data, or biological material

Policy information about studies with human participants or human data. See also policy information about sex, gender (identity/presentation), and sexual orientation and race, ethnicity and racism.

| | |
|---|---|
| Reporting on sex and gender | *Use the terms sex (biological attribute) and gender (shaped by social and cultural circumstances) carefully in order to avoid confusing both terms. Indicate if findings apply to only one sex or gender; describe whether sex and gender were considered in study design; whether sex and/or gender was determined based on self-reporting or assigned and methods used. Provide in the source data disaggregated sex and gender data, where this information has been collected, and if consent has been obtained for sharing of individual-level data; provide overall numbers in this Reporting Summary. Please state if this information has not been collected. Report sex- and gender-based analyses where performed, justify reasons for lack of sex- and gender-based analysis.* |
| Reporting on race, ethnicity, or other socially relevant groupings | *Please specify the socially constructed or socially relevant categorization variable(s) used in your manuscript and explain why they were used. Please note that such variables should not be used as proxies for other socially constructed/relevant variables (for example, race or ethnicity should not be used as a proxy for socioeconomic status). Provide clear definitions of the relevant terms used, how they were provided (by the participants/respondents, the researchers, or third parties), and the method(s) used to classify people into the different categories (e.g. self-report, census or administrative data, social media data, etc.) Please provide details about how you controlled for confounding variables in your analyses.* |
| Population characteristics | *Describe the covariate-relevant population characteristics of the human research participants (e.g. age, genotypic information, past and current diagnosis and treatment categories). If you filled out the behavioural & social sciences study design questions and have nothing to add here, write "See above."* |
| Recruitment | *Describe how participants were recruited. Outline any potential self-selection bias or other biases that may be present and how these are likely to impact results.* |
| Ethics oversight | *Identify the organization(s) that approved the study protocol.* |

Note that full information on the approval of the study protocol must also be provided in the manuscript.

# Field-specific reporting

Please select the one below that is the best fit for your research. If you are not sure, read the appropriate sections before making your selection.

☒ Life sciences ☐ Behavioural & social sciences ☐ Ecological, evolutionary & environmental sciences

For a reference copy of the document with all sections, see nature.com/documents/nr-reporting-summary-flat.pdf

# Life sciences study design

All studies must disclose on these points even when the disclosure is negative.

| | |
|---|---|
| Sample size | No statistical method was used to pre-determine the sample size. Sample sizes were chosen to assure reliable statistical difference and reproducibility and the selections were guided by the previous studies from the lab. |
| Data exclusions | No data was excluded from the study. |
| Replication | All experiments were replicated independently a minimum of three times and all replication attempts were successful. |
| Randomization | Animals were allocated in experimental groups randomly. |
| Blinding | The investigators were not blinded to experimental group assignments in animal, cellular or biochemical studies because the experiments needed several interventions (drug treatments etc.) by the investigators. |

# Reporting for specific materials, systems and methods

We require information from authors about some types of materials, experimental systems and methods used in many studies. Here, indicate whether each material, system or method listed is relevant to your study. If you are not sure if a list item applies to your research, read the appropriate section before selecting a response.

## Materials & experimental systems

| n/a | Involved in the study |
|---|---|
| ☐ | ☒ Antibodies |
| ☐ | ☒ Eukaryotic cell lines |
| ☒ | ☐ Palaeontology and archaeology |
| ☐ | ☒ Animals and other organisms |
| ☐ | ☒ Clinical data |
| ☒ | ☐ Dual use research of concern |
| ☒ | ☐ Plants |

## Methods

| n/a | Involved in the study |
|---|---|
| ☒ | ☐ ChIP-seq |
| ☐ | ☒ Flow cytometry |
| ☒ | ☐ MRI-based neuroimaging |

## Antibodies

Antibodies used

Primary and secondary antibodies used in the study were as follows:
Anti RTN3 (Abcam, ab187764, Western blotting [WB] 1/1000, Immunoflouorescence [IF] 1/250), Anti CD63 (BD Pharmingen, 551458, WB 1/1000, IF 1/250), Anti VDAC1/2 (Proteintech, 55259-1-AP, Abcam, ab14734, WB 1/2000), Anti Myc (Cell Signaling, 2272S, WB 1/1000) Anti Tubulin (Proteintech, 10094-1-AP, WB 1/5000), Anti GFP (Proteintech, 50430-2-AP, WB 1/4000), Anti SNAP47 (SYSY, 111403, WB 1/1000), Anti Syntaxin5 (Gift from Richard Scheller, WB 1/1000), Anti Syntaxin17 (Sigma-Aldrich, HPA001204, WB 1/1000), Anti GRP78/Bip (Proteintech, 11587-1-AP, WB 1/2000), Anti Phospho-eIF2α (Ser51) (Cell Signaling, D9G8, WB 1/1000), Total eIF2α (Cell Signaling, D7D3, WB 1/1000), GAPDH (Sigma-Aldrich, G9545, WB 1/10000), Anti p-S616 DRP1 (Cell Signaling, 4494S, WB 1/1000), Anti p-S637 DRP1 (Cell Signaling, 6319, WB 1/1000), Anti DRP1 (Cell Signaling, 8570S, WB 1/1000), Anti MFF (Cell Signaling, 84580 WB 1/1000), Anti OPA1 (BD Biosciences, 612606, WB 1/1000), Anti MFN2 (Abcam, ab124773, WB 1/1000), Anti Beta-Actin (Sigma-Aldrich, A5441, WB 1/20000), Anti pS2448-mTOR (Cell Signaling, 5536P, WB 1/1000), Anti mTOR (Cell Signaling, 2983P, WB 1/1000), Anti LC3B (Abcam, ab48394, WB 1/1000), Anti p62/SQSTM1 (Abcam, ab56416, WB 1/5000), Anti pS757-ULK1 (Cell Signaling, 14202T, WB 1/1000), Anti ULK1 (Cell Signaling, 8054, WB 1/1000), Anti pT389-P70S6K (Cell Signaling, 9208S, WB 1/1000), P70S6K (Cell Signaling, 9234, WB 1/1000), Anti-rabbit IgG, HRP-linked Antibody (Cell Signaling, 7074, WB 1/10000), Anti-Mouse IgG, HRP- linked Antibody (Cell Signaling, 7076, WB 1/10000), Alexa Fluor 568 Goat Anti-Mouse (ThermoScientific, A11031, IF 1/250), Alexa Fluor 568 Donkey Anti-Mouse (ThermoScientific, A10037, IF 1/250), Alexa Fluor 488 Donkey Anti-Rabbit (ThermoScientific, A21206, IF 1/250), Vimentin (Abcam ab92547, EPR3776, IHC 1/250), Iba1 (WAKO, 019-19741, IHC 1/800).

Validation

All the antibodies used in the study have been validated commercially. The STX5 antibody was verified in the prior publications of the Richard Scheller lab.

## Eukaryotic cell lines

Policy information about cell lines and Sex and Gender in Research

Cell line source(s)

American Type Culture Collection (ATCC)

Authentication

VAMP7KO and ATG5KO NRK and RG2 were generated in-house following the protocol described in our earlier publication (PMID:33357422). The loss of VAMP7 and ATG5 was verified using western blotting.

Mycoplasma contamination

All cell lines tested negative for mycoplasma contamination.

Commonly misidentified lines
(See ICLAC register)

None of the cell lines used in this study are listed as misidentifed lines in the ICLAC register.

## Animals and other research organisms

Policy information about studies involving animals; ARRIVE guidelines recommended for reporting animal research, and Sex and Gender in Research

Laboratory animals

Fisher F344 rats were obtained from Janvier Labs, France. We used 7-8 weeks old adult male rats in our studies. 2-3 rats were housed per cage with enrichment, 21°C, under a 12h/12h light/dark cycle, with water and food available ad libitum.

Wild animals

This study did not involve the use of wild animals.

Reporting on sex

We used only male rats in our studies to not introduce hormonal bias and its potential impact on glioblastoma progression.

| | |
|---|---|
| Field-collected samples | This study did not use any field collected samples |
| Ethics oversight | All the procedures were performed according to the European Communities Council (Directive 2010/63/EU) guildelines and were duly approved by the Ethical Commitee Comité d'Éthique en matière d'Expérimentation Animale Paris Descartes. |

Note that full information on the approval of the study protocol must also be provided in the manuscript.

## Clinical data

Policy information about clinical studies
All manuscripts should comply with the ICMJE guidelines for publication of clinical research and a completed CONSORT checklist must be included with all submissions.

| | |
|---|---|
| Clinical trial registration | *Provide the trial registration number from ClinicalTrials.gov or an equivalent agency.* |
| Study protocol | *Note where the full trial protocol can be accessed OR if not available, explain why.* |
| Data collection | *Describe the settings and locales of data collection, noting the time periods of recruitment and data collection.* |
| Outcomes | *Describe how you pre-defined primary and secondary outcome measures and how you assessed these measures.* |

## Plants

| | |
|---|---|
| Seed stocks | *Report on the source of all seed stocks or other plant material used. If applicable, state the seed stock centre and catalogue number. If plant specimens were collected from the field, describe the collection location, date and sampling procedures.* |
| Novel plant genotypes | *Describe the methods by which all novel plant genotypes were produced. This includes those generated by transgenic approaches, gene editing, chemical/radiation-based mutagenesis and hybridization. For transgenic lines, describe the transformation method, the number of independent lines analyzed and the generation upon which experiments were performed. For gene-edited lines, describe the editor used, the endogenous sequence targeted for editing, the targeting guide RNA sequence (if applicable) and how the editor was applied.* |
| Authentication | *Describe any authentication procedures for each seed stock used or novel genotype generated. Describe any experiments used to assess the effect of a mutation and, where applicable, how potential secondary effects (e.g. second site T-DNA insertions, mosiacism, off-target gene editing) were examined.* |

## Flow Cytometry

### Plots

Confirm that:

☒ The axis labels state the marker and fluorochrome used (e.g. CD4-FITC).

☒ The axis scales are clearly visible. Include numbers along axes only for bottom left plot of group (a 'group' is an analysis of identical markers).

☒ All plots are contour plots with outliers or pseudocolor plots.

☒ A numerical value for number of cells or percentage (with statistics) is provided.

### Methodology

| | |
|---|---|
| Sample preparation | Cells were incubated with 200 nM MitoTracker GreenTM for 30 min, followed by washing, trypsinization, centrifugation and resuspension in PBS + 0.2% FBS at cell concentration of 1000000 cells/mL |
| Instrument | BD FACSCaliburTM (BD Biosciences) |
| Software | BD CellQuest™ Pro Software Acquisition (for acquisition and analyses) and FlowJo (for visualization |
| Cell population abundance | At least 50000 events were acquired per experience. |
| Gating strategy | FCS-H vs SSC-H plots were used to define the gating, thereby excluding debris and very big cells. |

☒ Tick this box to confirm that a figure exemplifying the gating strategy is provided in the Supplementary Information.
